# The impact of relative deprivation on mental health among middle-aged and older adults in China: a multiple chain mediation model

**DOI:** 10.1038/s41598-025-20182-8

**Published:** 2025-09-30

**Authors:** Zhiruo Zhuang, Xinlong Xie, Longyi Huang, Jiawen Yan, Sijie Cheng, Aijun Xu

**Affiliations:** 1 School of Health Economics and Management, Nanjing University of Chinese Medicine, Nanjing, China; 2 Northern Jiangsu People’s Hospital, Yangzhou, China; 3 Jiangsu Research Center for Major Health Risk Management andTCM Control Policy, Nanjing University of Chinese Medicine, Nanjing, China

**Keywords:** Relative deprivation, Mental health, Social justice, Social trust, Multiple chain mediation, Psychology, Risk factors, Psychology and behaviour

## Abstract

This study, grounded in the theoretical frameworks of social comparison and institutional trust, employs a multiple mediation model to elucidate the mediating mechanisms of social justice and social trust in the relationship between relative deprivation and mental health. Using data from 3777middle-aged and older adults in the Chinese General Social Survey, we conducted OLS regression and bootstrap analyses. The results demonstrate that relative deprivation among middle-aged and older adults not only is negatively correlated with mental health, but is also indirectly associated with mental health through two mediating variables: social justice and social trust. Furthermore, bootstrap analysis reveals a significant serial mediation pathway from relative deprivation through social justice to social trust, with an effect size of −0.002. These findings suggest that interventions targeting reduced relative deprivation, enhanced perceptions of social justice, and strengthened social trust may effectively improve mental health outcomes in this population.

## Introduction

According to a recent meta-analysis, late-life depression has been identified as a major public health concern in China. As of 2021, the prevalence of depressive disorders among older adults was approximately 20%, with data indicating a continuing upward trend^1^. Concurrently, China’s population aging is accelerating, with the seventh national census revealing that individuals aged ≥ 45 years exceeded 500 million, accounting for 32.11% of the total population. The mental health status of this large group not only directly affects their quality of life, but also influences familial well-being and social stability. However, due to the dual impact of social transformation and aging pressure, mental health issues among middle-aged and older people have become increasingly prominent. The high prevalence of psychological disorders, such as anxiety and depression, has emerged as a significant social concern that warrants attention^2^. In this context, exploring the mechanisms influencing the mental health of middle-aged and older adults and formulating targeted intervention strategies are not only urgent necessities for enhancing personal well-being but also critical measures for achieving healthy aging and ensuring sustainable social development. Research indicates a significant association between socioeconomic status (SES) and mental health^3–5^ with relative deprivation, as an important subjective dimension of SES, exerting a particularly pronounced influence on mental health^6,7^.

Relative deprivation refers to a subjective cognitive and emotional experience in which individuals or groups perceive themselves to be at a disadvantage by comparing themselves with a reference group. As a key mechanism in health inequality research, this concept draws on theoretical origins in Social Comparison Theory (SCT)^8^ and Fundamental Cause Theory (FCT)^9^. SCT elucidates how individuals develop perceptions of deprivation through horizontal or vertical comparisons^10^ while FCT further posits that such perceptions essentially reflect how socioeconomic status, as a fundamental social determinant, perpetuates health inequalities through dynamically evolving multiple pathways. Together, these theories explain why middle-aged and older adults are particularly vulnerable to relative deprivation. As they experience role transitions and physiological decline, they encounter not only a reduced capacity to acquire objective resources but also a reconstruction of social reference frames, which can amplify subjective disadvantage. This dual mechanism renders them more susceptible to the cumulative effects of deprivation, triggering depression^11,^ anxiety disorders^12,^ and aggressive behaviors^13^. Although prior research has primarily focused on the direct relationship between relative deprivation and adverse psychological states^14,15^ the underlying mediating mechanisms remain unclear. Therefore, building upon FCT, this study proposes a serial multiple mediation model to uncover the correlation path between relative deprivation and mental health in middle-aged and older adults. Specifically, we examine social justice and social trust as mediators to analyze the mechanisms by which relative deprivation affects psychological well-being in this demographic.

## Relative deprivation and mental health

According to classical Relative Deprivation Theory (RDT)^13,^ individuals evaluate their own situation and status primarily through comparisons with others. Members of disadvantaged groups may develop a perception that their entitled resources are being deprived by other groups, and this sense of deprivation can severely harm their psychological and physical well-being^16^.

Previous studies have demonstrated that relative deprivation is not only directly associated with mental health^17,18^ but may also indirectly affect mental health through other mediating variables^19^. First, grounded in Social Comparison Theory, relative deprivation adversely impacts mental health by eliciting negative emotions^18^. Research by Osborne et al. demonstrated that individual relative deprivation is significantly negatively associated with self-reported mental health, with emotions such as anger, fear, and sadness playing a mediating role in this relationship^20^. Research indicates that individuals of lower social status are more likely to experience relative deprivation^21^ which subsequently heightens the risk of depression and anxiety. Furthermore, the relationship between relative deprivation and mental health problems is more pronounced in regions characterized by greater socioeconomic inequality^22^. Second, perceptions of relative deprivation affect mental health by weakening social support systems. Research indicates that individuals who perceive relative deprivation often exhibit a diminished sense of trust in society^23^ and encounter challenges in forming and sustaining positive social support networks, thereby exacerbating mental health issues. Additionally, relative deprivation may correlate with social isolation by diminishing individuals’ willingness to participate in social activities^24^ thereby undermining their psychological resilience. Finally, relative deprivation indirectly affects mental health by influencing an individual’s health behaviors. Research suggests that relative deprivation is associated with unhealthy lifestyle choices^25^ such as smoking, alcohol abuse, and physical inactivity^26^. These behaviors not only compromise physical health but also adversely impact mental health^27^. In summary, relative deprivation negatively affects mental health through various mechanisms, including triggering negative emotions, weakening social support, and influencing healthy behaviors.

### Social justice as a mediator

Based on Runciman’s theory of relative deprivation, when individuals perceive an unfair distribution of resources in society, their sense of relative deprivation significantly increases. This heightened perception, in turn, accumulates negative emotions and jeopardizes mental health^28^. The applicability of this theory to the middle-aged and older population is supported by empirical research. Smith^13^ demonstrated through a meta-analysis that a sense of social fairness significantly mediates the relationship between relative deprivation and mental health impairment. Furthermore, the negative impact of relative deprivation on mental health can be substantially mitigated when individuals possess a positive assessment of the justice of resource allocation. Zhang et al.^29^ found that a mediated sense of social justice in essential livelihood sectors such as healthcare and elder care, can mitigate the effects of relative deprivation on the mental health of middle-aged and older adults^30^. For this demographic, the specificity of their life stage—characterized by factors such as income disparity due to retirement and increased medical dependence resulting from declining health—significantly heightens their sensitivity to relative deprivation^31^. Consequently, any micro-level perception of disparity may be transformed into a sense of injustice through the attribution amplification mechanism, thereby establishing a chain reaction path of ‘relative deprivation→ perceived injustice → psychological exhaustion’^2^. In view of this, enhancing the sense of social justice not only effectively mitigates the psychologically damaging effects of relative deprivation, but also serves as a crucial policy target for improving the mental health of middle-aged and older adults.

### Social trust as a mediator

Social trust, defined as an individual’s generalized belief in the trustworthiness and benevolence of others^32,^ has been identified as a critical mediator between relative deprivation and mental health^33^. According to FCT, SES represents a “fundamental” determinant of health, shaping one’s access to health-protective resources such as knowledge, power, and social connections^9^. Relative deprivation, as a subjective perception of one’s disadvantaged SES, can trigger a similar pathway. When middle-aged and older adults develop a strong sense of deprivation due to income, status, or intergenerational comparisons, this cognitive bias may generalize into a broad insecurity and skepticism toward the external world—including other people—thereby eroding social trust^34^. From the perspective of SCT^35,^ social trust constitutes a core component of social capital, which contributes to health by facilitating reciprocal cooperation and social support. High levels of social trust can buffer psychological stress and enhance a sense of belonging, both of which serve as important protective factors for mental health^36,37^. Conversely, a lack of social trust can undermine an individual’s social support network, leading to social withdrawal, loneliness, and feelings of powerlessness, thereby exacerbating emotional disorders such as depression and anxiety^38^.

Therefore, among middle-aged and older adults, relative deprivation may undermine social trust through the mechanism of resource deprivation as outlined by the FCT. Impaired social trust, in turn, fails to fulfill the health-promoting functions emphasized by SCT, ultimately resulting in poorer mental health outcomes.

### Social justice and social trust

Social justice enhances social trust by reducing interpersonal heterogeneity and disparities, thereby narrowing the social distance among individuals^39^. Tyler^40^ found that both procedural fairness (e.g., policy transparency, democratic participation) and distributive fairness (e.g., income disparity) directly affect individuals’ trust in social institutions, especially in the area of public policy. Alesina and La Ferrara^41^ assert that fairness in domains such as education and employment contributes to social trust, extending beyond mere economic indicators. Smith^13^ discovered that middle-aged and older adults exhibit heightened sensitivity to social justice issues and are more profoundly impacted by perceptions of social justice compared to their younger counterparts. Furthermore, a simulation experiment conducted by Neumark^42^ revealed that job seekers aged over 50 are 40% less likely to secure an interview. Such instances of ‘age discrimination’ were found to diminish social trust by 10–15%. This indicates that age discrimination exacerbates trust crises by undermining equitable opportunities. The decline in health has rendered middle-aged and older adults extremely sensitive to justice in the distribution of healthcare resources. Disparities in healthcare reimbursement between urban and rural areas have resulted in a diminished perception of justice within the healthcare system among rural older adults^43,^ who exhibit lower levels of social trust compared to their urban counterparts^44^. Collectively, these findings reinforce the central tenet of institutional trust theory, which posits that perceived social justice is a crucial predictor of social trust.

With the physiological aging and social role transformation experienced by middle-aged and older adults, both horizontal comparisons with the external world and vertical comparisons with their past selves can evoke a sense of relative deprivation. This perception not only triggers emotional stress but also diminishes evaluation of social justice due to attribution bias^45^. When middle-aged and older adults perceive social rules as unjust, their trust in public systems and societal members systematically deteriorates, undermining their social relationships and support networks, which further weakens their mental health. Previous studies have identified the relationship between relative deprivation and mental health^46^ with some scholars suggesting that relative deprivation is most effectively transmitted through perceptions of social justice^47^. A lack of perceived justice can erode an individual’s social trust^48^ subsequently exerting a negative impact on mental health. However, researchers have rarely explored the pathways through which these variables interact and have often overlooked the specificities of middle-aged and older adults.

### The current study

Previous studies have primarily focused on the antecedents of relative deprivation—such as individual attribution styles^49^ demographic characteristics^50^ and socio-environmental variables^51^—as well as its outcomes, including health^16^ cognition^52,^ and behavior^26,50^. However, research on the underlying mediating mechanisms remains fragmented and lacks an integrated theoretical framework to elucidate the pathways through which these effects occur.

This study innovatively integrates the FCT and SCT to examine the interlinking factors affecting mental health among middle-aged and older adults in China. Specifically, it investigates the serial multiple mediation effects of perceived social justice and social trust in the relationship between relative deprivation and mental health in this population. By testing these relationships, we seek to gain a comprehensive understanding of how relative deprivation correlates with mental health issues. In this study, we propose the following hypotheses:

#### H1

Relative deprivation among middle-aged and older adults is negatively associated with their level of mental health.

#### H2

Social justice and social trust mediate the associations between relative deprivation and mental health, respectively.

#### H3

Social justice and social trust exhibit multiple chain mediation effects between relative deprivation and mental health.

## Methods

### Data source

**Fig. 1:**
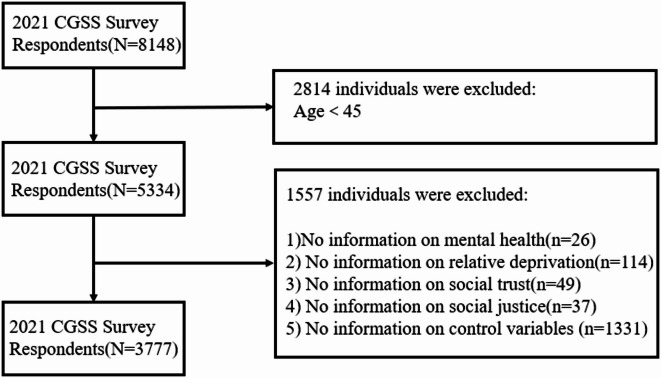
Respondents were included in the flowchart.

In this paper, the 2021 resident questionnaire data from the Chinese General Social Survey (CGSS) were selected as the most recent available for the study. CGSS2021 was publicly released on March 31, 2023. The survey employed multi-stage stratified sampling to select samples from 28 provinces, autonomous regions, and municipalities in China, resulting in a total sample size of 8148. After deleting missing values and outliers, 3777 valid observations were retained. The specific incorporation process is shown in Fig. 1.

### Measures

#### Dependent variable: mental health

Mental health as the dependent variable in this paper, referring to previous studies^2,53–56^ and the questionnaire design, we used the question “In the past four weeks, how often have you felt depressed or down? (always, often, sometimes, rarely, never)”, and assigned values from 1 to 5, with lower scores representing lower levels of mental health.

#### Independent variable: relative deprivation

For the measurement of relative deprivation, drawing upon prior studies^47,57,58^ and grounded in the core tenets of Social Comparison Theory and Relative Deprivation Theory, we adopted the classic subjective socioeconomic status measure from CGSS2021: “Overall, in current society, where would you place your own socioeconomic status?” Respondents selected from five tiers (upper, upper-middle, middle, lower-middle, lower), with higher tiers indicating lower perceived relative deprivation. Compared to objective indicators, subjective SES—a comprehensive assessment of an individual’s social standing derived from social comparisons—more sensitively captures an individual’s perceived relative position in their social reference groups. This is because it reflects not only actual material resource ownership but also incorporates multidimensional comparisons—including cultural capital and social esteem. Within China’s collectivist context, such a subjective assessment is particularly effective in capturing comprehensive perceptions of disadvantage stemming from structural inequalities. While relative deprivation is multidimensional, the foundational role of socioeconomic status is well-established in academia^59^. In this study, relative deprivation specifically refers to subjective socioeconomic deprivation.

#### Mediating variables: social justice and social trust

One of the mediating variables in this paper is residents’ subjective perception of social justice. The corresponding survey question was: “Do you think today’s society is just?” Respondents were given five choices, where 1 to 5 represented “not at all just” to “completely just”. Higher values indicated higher perceived social justice^60^.

The other mediating variable is social trust, defined as generalized trust in others. Although the standard question “In general, do you think most people can be trusted?”^61^ has been criticized as contextually vague, it remains valid for measuring abstract trust. The CGSS 2021 measured this construct with the locally adapted question: “Do you think most people in this society are trustworthy?” This phrasing was more culturally accessible for Chinese respondents^62^. Responses used a 5-point Likert scale from “totally disagree” (1) to “totally agree” (5), with higher scores denoting greater social trust^62,63^.

### Control variables

In order to control for confounding factors that may simultaneously affect relative deprivation and mental health, we screened control variables from multiple dimensions such as population structure, objective socio-economic status, social support network, and baseline health status. These variables include age(calculated as 2025 minus the year of birth), gender (*Ref.* = male), current residence (*Ref.* = town), political profile (*Ref.* = masses), hukou (a system of household registration in China, *Ref.* = Non-agricultural), marital status (*Ref.* = having a spouse), household size, religious affiliation (*Ref.* = yes), self-assessed health status(from very unhealthy = 1 to very healthy = 5, higher values = better self-assessed health status), insurance participation (*Ref.* = yes), employment status (*Ref.* = working), etc.

### Statistical analysis

This study constructed an initial theoretical model based on Social Comparison Theory and Fundamental Cause Theory, with statistical analyses performed using SPSS 25.0. First, descriptive statistics were employed to examine the general characteristics of the study population. Continuous variables were presented as means and standard deviations, while categorical variables were expressed as frequencies and percentages. Second, Pearson product-moment correlation analysis was conducted to assess the strength of associations among variables including relative deprivation, social justice, social trust, and mental health. Subsequently, hierarchical linear regression analysis was performed to clarify causal pathways between variables. Demographic covariates such as age, gender, and education level were included, and the direct effects of the independent variable (relative deprivation) and the mediating variables (social justice and social trust) on the dependent variable (mental health) were sequentially tested. Next, Model 6 of Hayes’ PROCESS v4.1 macro^64^ was used to examine serial mediation effects. The multiple serial mediation model allows exploration of mechanisms whereby independent variables sequentially influence dependent variables through ordered mediators, and its applicability in Chinese cultural contexts has been empirically demonstrated^65^. This model was selected because it aligns with the theoretical framework analyzing how structural social factors (e.g., SES) influence individual health through multi-layered socio-cognitive pathways. Finally, the significance of mediation effects was tested using the Bootstrap resampling method (1000 repetitions) at *P* < 0.05. A statistically significant effect was indicated if the 95% bias-corrected bootstrap confidence interval (95% CI) of the indirect effect did not include zero.

## Results

### Descriptive statistics

Table 1 presents a descriptive statistical analysis of all of the variables.

**Table 1:** Definition and descriptive statistics of various variables.

Variables	Definitions	Mean/Frequency	SD/Percentage
Relative deprivation	Upper = 1; Upper middle = 2; Middle = 3; Lower middle = 4; Lower = 5 (higher values = more relative deprivation)	3.704	0.935
Social trust	Strongly disagree = 1; Comparatively disagree = 2; Can’t say whether I agree or disagree = 3; Comparatively agree = 4; Strongly agree = 5	3.762	0.980
Social justice	Very unfair = 1; More unfair = 2; Can’t say if it’s fair or unfair = 3; Fairer = 4; Very fair = 5	3.527	0.986
Mental health	Frequency of depression : Always = 1; Often = 2; Sometimes = 3; Rarely = 4; Never = 5; (higher values = better mental health)	3.958	1.101
Gender	Male = 1	1946	52.52%
	Female = 2	1831	48.485
Age	Continuous variable: Year	62.083	10.405
Ethnicity	Han = 1	3561	94.28%
	Otherwise = 0	216	5.72%
Education	Below junior high school = 1	2727	72.20%
	High school = 2	709	18.77%
	College and Bachelor’s Degree = 3	333	8.82%
	Undergraduate or higher = 4	8	0.21%
Religious affiliation	Yes = 1	283	7.49%
	No = 0	3494	92.51%
Political profile	Masses = 1	3176	84.09%
	Democrats = 2	3	0.08%
	Communists = 3	598	15.83%
Current residence	Neighborhood committee = 1	2452	64.92%
	Village committee = 2	1325	35.085
Household size	Concrete figure	2.278	1.882
Marital status	Having a spouse = 1	3084	81.65%
	No spouse = 0	693	18.35%
Employment situation	Working = 1	1849	48.95%
	Unemployed = 0	1928	51.05%
Self-rated health	Very unhealthy = 1; Relatively unhealthy = 2; Generally healthy 3; Relatively healthy = 4; Very healthy = 5	3.282	1.079
Hukou	Non-agricultural = 1	1722	45.59%
	Agriculture = 2	2055	54.41%
Old-age insurance	Yes = 1	3132	82.92%
	No = 0	645	17.08
Medical insurance	Yes = 1	3601	95.34%
	No = 0	176	4.66%
Real estate	Concrete figure	1.230	0.844
Incomes	Logarithms	4.253	0.590

### Correlation between major variables

In Table 2, the results of Pearson’s correlation analysis indicate statistically significant correlations between relative deprivation, social justice, social trust, and the mental health of middle-aged and older adults. Specifically, mental health is positively correlated with social justice (*r* = 0.124, *p* < 0.001) and social trust (*r* = 0.110, *p* < 0.001), while it is negatively correlated with relative deprivation (*r*= − 0.205, *p* < 0.001). Furthermore, relative deprivation shows a negative correlation with both social justice (*r*= − 0.205, *p* < 0.001) and social trust (*r*= − 0.108, *p* < 0.001). Additionally, social justice was positively correlated with social trust (*r* = 0.331, *p* < 0.001).

**Table 2:** Correlations among key variables.

	Mental health	Relative deprivation	Social justice	Social trust
Mental health	1.000			
Relative deprivation	− 0.205***	1.000		
Social justice	0.124***	− 0.205***	1.000	
Social trust	0.110***	− 0.108***	0.331***	1.000

*p < 0.05, **p  < 0.01, ***p < 0.001

### Mediation analysis of social justice and social trust

This study examined the relationship between relative deprivation and mental health of middle-aged and older adults, controlling for several potential influencing factors, including gender, age, type of residence, marital status, and income. The analysis of the data presented in Table 3 revealed that relative deprivation significantly negatively impacted the mental health of middle-aged and older adults (β = − 0.084, *p* < 0.001). Conversely, both social justice (β = 0.058, *p* < 0.001) and social trust (β = 0.069, *p* < 0.001) demonstrated significant positive effects on the mental health of this demographic.

**Table 3:** Hierarchical linear regression analysis of the impact of relative deprivation on mental Health.

Variables	Model: Mental health
	β (SE)
Relative deprivation	− 0.084^***^(0.018)
Social justice	0.058^***^(0.017)
Social trust	0.069^***^(0.017)
Gender	− 0.163^***^ (0.033)
Age	0.004^*^(0.002)
Ethnicity	0.007(0.070)
Education	0.002(0.029)
Religious affiliation	− 0.096(0.061)
Political profile	0.040 (0.016)
Hukou	− 0.136^**^(0.045)
Marital status	0.184^***^(0.043)
Current residence	− 0.156^***^(0.042)
Household size	0.025^**^(0.009)
Employment situation	0.022(0.039)
Self-rated health	0.370^***^(0.016)
Old-age insurance	0.053(0.043)
Medical insurance	− 0.064(0.077)
Real estate	− 0.004(0.019)
Incomes	0.105^**^(0.036)
Constant	2.285^***^(0.317)
Observations	3777
R-squared	0.238
Adjusted R-squared	0.234

*p < 0.05, **p < 0.01, ***p < 0.001; standard errors in parentheses; Due to spatial limitations, we only presented the final model graph that includes all variables.

### Bootstrap mediator path checking

As shown in Table 4, none of the 95% confidence intervals (CIs) for relative deprivation and mental health included zero when mediated solely through social justice or social trust, indicating that the indirect effects were significant (indirect effect of social justice = −0.011; indirect effect of social trust = − 0.007). Social justice, acting as a mediator between relative deprivation and mental health, accounted for 10.68% (−0.011/−0.103) of the variance in mental health. Similarly, social trust as a mediator between relative deprivation and mental health explained 6.80% (−0.007/−0.103) of the variance in mental health. The third indirect path reveals that the effect of relative deprivation on mental health is significantly mediated by both social justice and social trust, with an indirect effect value of −0.002, elucidating 1.94% (−0.002/−0.103) of the variance in mental health. Consequently, the Bootstrap test results (1000 repetitions) corroborated the partial mediating effects of social justice and social trust in the relationship between relative deprivation and mental health among middle-aged and older adults, collectively elucidating 18.45% (−0.019/−0.103) of the variance in mental health levels. These findings imply that relative deprivation may partially affect mental health by undermining perceptions of institutional fairness and relational security.

**Fig. 2:**
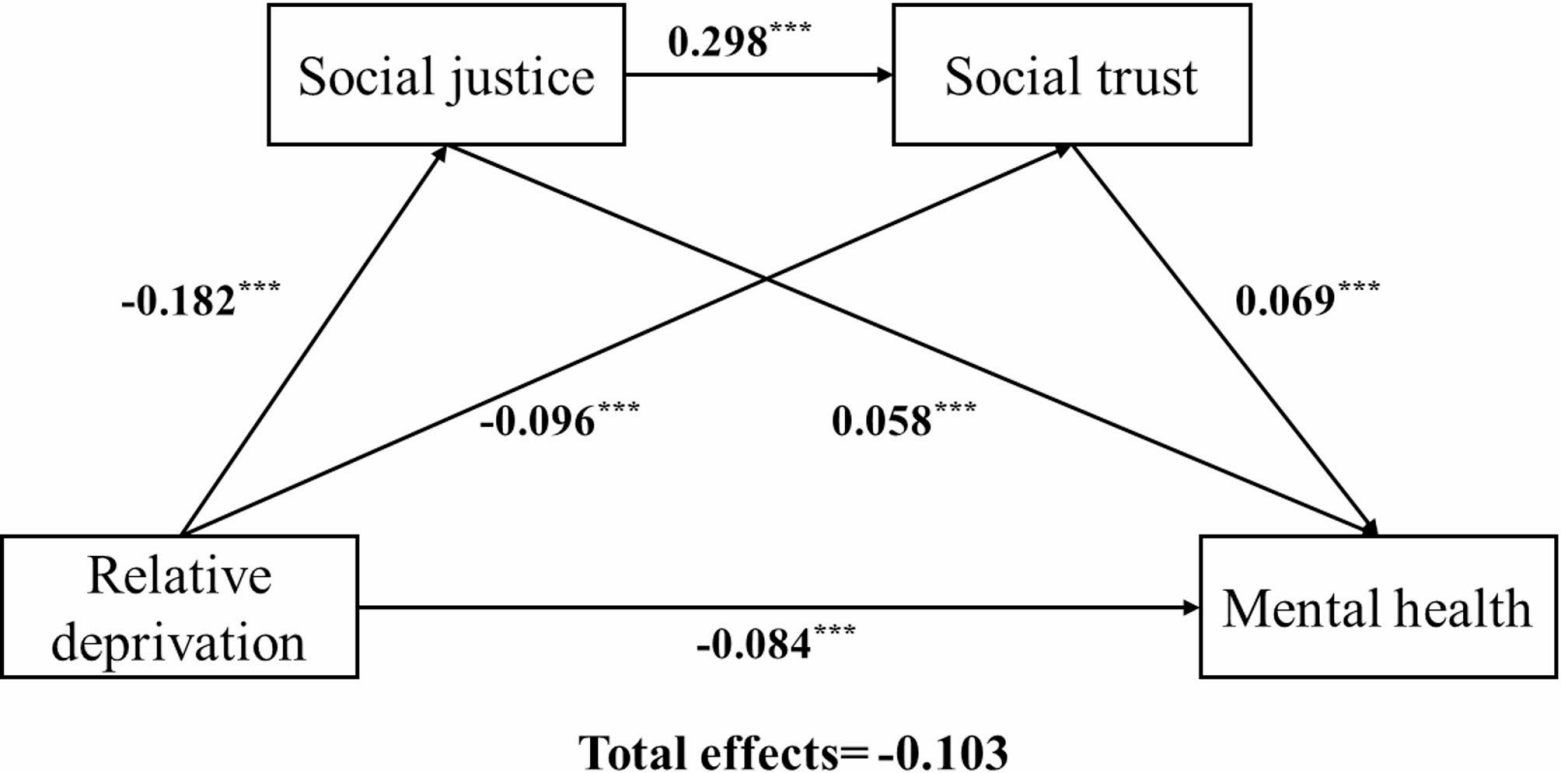
Serial mediation models for relative deprivation, mental health, social justice and social trust.* Note* path coefficients were shown in standardized regression coefficient. ***p < 0.001.

Figure 2 and Table 4 jointly present the results of the serial mediation analysis. In Fig. 2, solid arrows with numerical values indicate statistically significant path coefficients, while the corresponding Bootstrap test results are reported in Table 4.

**Table 4:** Bootstrap analysis of the mediated effects test.

Pathway	Effect	SE	Boot LLCI	Boot ULCI	Effect ratio (%)
Total direct effect	− 0.084	0.018	− 0.120	− 0.048	81.55
Total mediation effect	− 0.019	0.004	− 0.023	− 0.009	18.45
Relative deprivation → social justice → mental health	− 0.011	0.003	− 0.014	− 0.003	10.68
Relative deprivation → social trust → mental health	− 0.007	0.002	− 0.010	− 0.002	6.80
Relative deprivation → social justice → social trust → mental health	− 0.002	0.001	− 0.003	− 0.000	1.94
Total effect	− 0.103	0.018	− 0.138	− 0.067	100.00

Standardized estimates of 1000 bootstrap samples. Boot LLCI: lower level of the 95% confidence interval; Boot ULCI: upper level of the 95% confidence interval; SE, standard error; Effect, standardized regression coefficient.

## Discussion

The primary objective of this study was to examine the mediating roles of social justice and social trust in the relationship between relative deprivation and mental health among middle-aged and older Chinese adults. Firstly, relative deprivation among middle-aged and older adults is negatively associated with their level of mental health. Secondly, both social justice and social trust were found to partially mediate the relationship between relative deprivation and mental health. Finally, social justice and social trust exhibited multiple chain-mediated effects between relative deprivation and mental health. Therefore, all hypotheses in the previous section have been substantiated.

Consistent with previous studies^15,66,^ we found that relative deprivation was negatively associated with mental health among middle-aged and older Chinese, accounting for more than 4/5 of the total variance in mental health. According to social comparison theory, feelings of subordination or inferiority stemming from relative deprivation can induce depression^67^. Individuals with low subjective SES may experience greater economic stress compared to those with higher subjective SES, generating feelings of frustration and a persistent sense of being belittled^68,^ which can lead to poor mental health outcomes. From a cognitive model of depression, relative deprivation significantly contributes to cognitive vulnerability regarding health^16^ and can create a profound sense of despair, directly resulting in depression^69^. Furthermore, individuals who experience strong relative deprivation over extended periods often develop adverse emotional responses^69^ such as anger, anxiety, and depression, with the accumulation of these emotions being a major factor in the deterioration of mental health.

Based on a multiple mediation model, we demonstrate that the indirect effects between relative deprivation and mental health among middle-aged and older Chinese adults can be mediated by social justice and social trust. These findings align with FCT’s “principle of multiple mechanisms”: perceived social justice reflects institutional resource allocation (e.g., healthcare accessibility), while social trust represents informal support networks (e.g., neighborhood mutual aid). Together, these factors constitute the operational pathways of fundamental causes in the Chinese context. Regarding social justice, our results suggest that middle-aged and older adults who experience a strong sense of relative deprivation tend to evaluate social justice negatively, which in turn leads to a decline in mental health. Previous studies have indicated a negative correlation between feelings of relative deprivation and perceptions of social justice^13^. According to the ‘structural determinism’ theory of social justice, individuals with higher SES generally exhibit lower levels of relative deprivation and are therefore more likely to endorse the current distributional situation. Conversely, those with lower SES are more inclined to perceive the existing distribution of resources as unjust^70^.The widening perception of injustice contributes to an increased likelihood of mental health issues among middle-aged and older adults^71^. Research indicates that social trust is negatively correlated with relative deprivation, which subsequently leads to a decline in mental health. Previous studies have demonstrated that relative deprivation, along with negative emotions and poor social perceptions among middle-aged and older adults, may result in a diminished level of social trust^72^ and even undermine government credibility. Individuals with low social trust tend to report feelings of alienation, insufficient social support, and a reduced sense of security. These factors increase their vulnerability to stress, loneliness, and depression, thereby elevating the risk of mental health disorders^73^.

In addition to examining the independent mediating role of social justice and social trust, we investigated the potential chain mediating effect between relative deprivation and mental health. The findings of this study are consistent with the FCT, indicating that relative deprivation is initially negatively associated with perceived social justice, which subsequently diminishes levels of social trust, and ultimately serves as a predictive factor for mental health issues. Previous research by Lu et al.^75^ demonstrated that increased relative deprivation significantly reduces perceived social justice and social trust levels. Lower perceived social justice within a community makes it more challenging to establish social trust^28^. Furthermore, low trust can provoke hostility during social comparisons, reduce inter-individual cooperation and mutual support behaviors, and heighten individual anxiety regarding future uncertainty^75^. These mechanisms collectively contribute to poorer mental health outcomes.

Although the social justice and social trust pathways account for 18.45% of the variance, the FCT highlights the necessity of addressing more systematic pathogenic networks. Substantial empirical evidence indicates that chronic stress and social exclusion represent significant unmeasured complementary pathways. Specifically, prolonged socioeconomic disadvantage—a state of scarcity of resources and opportunities due to low SES—persistently activates the hypothalamic-pituitary-adrenal (HPA) axis. This activation results in down-regulated glucocorticoid receptor expression, a biological mechanism that independently contributes to depressive symptoms^76^. Concurrently, spatial exclusion mechanisms exacerbate psychological risks by constraining social capital accumulation^77^. When individuals become ensnared in the vicious cycle of ‘stress-resource deprivation’, multi-level intervention strategies demonstrate clear efficacy: Arkansas Medicaid expansion increased antidepressant treatment continuity by 20.5%^78^; China’s New Rural Cooperative Medical Scheme significantly reduced loneliness, anxiety, and depression among older adults^79^; and randomized controlled trials of community mutual-aid interventions showed a 73–76% reduction in suicide rates among older adults^80^. This integrated biomedical and psychosocial intervention approach fully aligns with the ‘primacy for structural interventions’ principle advocated by the WHO Commission on Social Determinants of Health^81^.

The selection of reference groups in measuring subjective SES has significant theoretical implications. Grounded in SCT^8^ and Park’s research^18,^ this study posits that subjective SES inherently reflects individuals’ “default reference framework” within specific social contexts. Against the backdrop of China’s social transformation, this reference framework exhibits distinct spatial, intergenerational, and institutional nesting characteristics. Spatially, urban and rural residents show marked divergence in their reference standards: rural populations predominantly use county-level economic development as their primary benchmark, while urban residents focus more on inter-city comparisons within provincial boundaries^82^. Generationally, new-generation migrant workers tend to compare themselves with urban residents, whereas older-generation migrants maintain stronger reference ties to rural hometown populations^83^. Additionally, China’s unique institutional segmentation has led to differentiated reference systems between public-sector and private-sector groups^84^. Although the five-tier subjective SES ladder measure employed in this study cannot directly quantify the specific contributions of each reference dimension, methodological controls for key variables (urban/rural residence, age, and occupation type) effectively mitigate potential estimation biases arising from reference group heterogeneity. Future research could enhance this measurement dimension by incorporating targeted questions.

While previous studies have established the association between relative deprivation and mental health across diverse populations, this study is the first to reveal a serial mediation pathway of “relative deprivation → social justice → social trust → mental health.” By innovatively integrating Fundamental Cause Theory, Social Comparison Theory, Institutional Trust Theory, and Relative Deprivation Theory within the framework of aging health research, our findings provide a theoretical foundation for enhancing mental well-being among middle-aged and older adults.

This study has several important limitations that must be acknowledged. Due to constraints in the database’s variable design, we were unable to employ established standardized scales (e.g., the Yitzhaki Index for relative deprivation and WHO-5 for mental health assessment) and had to rely on simplified single-item measures, which may affect the precision of effect size estimations between variables. The cross-sectional nature of the CGSS2021 data precludes the definitive establishment of causal sequencing or tracking of dynamic interactions among key variables. Although we conducted robustness checks using the Bootstrap method, the unidimensional nature of our measures (e.g., not distinguishing procedural justice from distributive justice) may weaken theoretical interpretability. These limitations reflect inherent trade-offs between measurement depth and breadth in large-scale social surveys. Future research should incorporate longitudinal data with standardized scales to further validate the long-term effects of these psychological mechanisms. We recommend interpreting these findings as exploratory evidence of psychosocial mechanisms among China’s middle-aged and older population rather than as definitive conclusions.

## Conclusion

This study provides an expanded theoretical lens for understanding the effects of relative deprivation. The findings demonstrate that, as a subjective evaluation of social status, relative deprivation is negatively correlated with mental health by eroding individuals’ beliefs in both the macro-environment (social justice) and micro-level interpersonal contexts (social trust). Consequently, it deprives individuals of two critical resources essential for psychological well-being. These results reinforce and extend the theoretical framework of social determinants of health, underscoring the need for integrated interventions that address both psychosocial and relational mechanisms. Based on empirical findings, we propose a three-tier intervention strategy: at the institutional level, implementing dynamic pension adjustment mechanisms linked to price indices to enhance systemic stability; at the governance level, establishing policy hearing systems for older adults to improve participatory efficacy through procedural fairness; and at the service level, integrating mental health services into essential public health packages while focusing on the development of community mutual-support networks. Collectively, these measures form a systemic solution to block the transmission of deprivation, with the core objective being the reconstruction of beliefs in social justice through institutional safeguards and the cultivation of a culture of generalized trust via participatory mechanisms. We recommend establishing a dynamic monitoring system that incorporates indicators such as relative deprivation indices and service accessibility to provide evidence-based support for policy optimization.

## Data Availability

Publicly available datasets were analyzed in this study. This data can be found here: [http://www.cnsda.org/index.php? r=projects/view&id=62072446](http:/www.cnsda.org/index.php?r=projects/view&id=62072446).
